# OSAS Surgery and Postoperative Discomfort: Phase I Surgery versus Phase II Surgery

**DOI:** 10.1155/2015/439847

**Published:** 2015-01-28

**Authors:** Giulio Gasparini, Andrea Torroni, Francesco Di Nardo, Sandro Pelo, Enrico Foresta, Roberto Boniello, Mario Romandini, Daniele Cervelli, Camillo Azzuni, Tito Matteo Marianetti

**Affiliations:** ^1^Maxillo Facial Surgery, School of Medicine, Catholic University of the Sacred Heart, 00168 Rome, Italy; ^2^Maxillo Facial Surgery, Complesso Integrato Columbus, 00168 Rome, Italy; ^3^Institute of Hygiene, School of Medicine, Catholic University of the Sacred Heart, 00168 Rome, Italy

## Abstract

*Introduction*. This study aims to investigate the reasons that discourage the patients affected by OSAS to undergo orthognathic surgery and compares the postoperative discomfort of phase I (soft tissue surgery) and phase II (orthognathic surgery) procedures for treatment of OSAS.* Material and Methods*. A pool of 46 patients affected by OSAS was divided into two groups: “surgery patients” who accepted surgical treatments of their condition and “no surgery patients” who refused surgical procedures. The “surgery patients” group was further subdivided into two arms: patients who accepted phase I procedures (IP) and those who accepted phase II (IIP). To better understand the motivations behind the refusal of II phase procedures, we asked the patients belonging to both the IP group and “no surgery” group to indicate the main reason that influenced their decision to avoid II phase procedures. We also monitored and compared five parameters of postoperative discomfort: pain, painkiller assumption, length of hospitalization, foreign body sensation, and diet assumption following IP and IIP procedures.* Results*. The main reason to avoid IIP procedures was the concern of a more severe postoperative discomfort. Comparison of the postoperative discomfort following IP versus IIP procedures showed that the former scored worse in 4 out of 5 parameters analyzed.* Conclusion*. IIP procedures produce less postoperative discomfort. IIP procedures, namely, orthognathic surgery, should be the first choice intervention in patients affected by OSAS and dentoskeletal malformation.

## 1. Introduction

Surgical procedures to treat obstructive sleep apnea syndrome (OSAS) aim either to debulk the soft tissues or to expand the skeletal frame; the former procedures aim to reduce/remove the obstructions due to the excessive bulk of soft tissues lining the rhinoorohypopharynx and may be performed as single or combined procedures, depending on patient exigencies.

Surgical procedures on soft tissue are generally defined as “first phase interventions” (IP) and aim to debulk soft tissues while maintaining the same skeletal volume [1–6].

When IP procedures fail in obtaining satisfactory results, “second phase interventions” (IIP) of orthognathic surgery are performed in order to increase the skeletal volume of the pharynx and stretching the soft tissues, producing, as final result, an effective enlargement of the air column.

There is general agreement in literature that the most effective and reliable interventions are the IIP [7–15]; nevertheless, as indicated by the nomenclature, patients often undergo IIP procedures only after failure of IP interventions, in total contradiction with what is reported.

We think that the reticence of physicians to propose IIP surgery depends fundamentally on their low familiarity with risks and complications of the orthognathic surgery, which results in deviant information and negative influence on patient's decisions.

The objective of this study is to identify the real motivations that discourage the patients to undergo IIP interventions and try to objectivize the discomfort following IP surgery compared with IIP procedures.

## 2. Materials and Methods

Forty-six patients affected by OSAS of various degrees have been seen in our clinic between January 1, 2008, and December 31, 2012. The sample included 26 males (56.5%); the mean age was 44 years (range 18–82, standard deviation (SD): 16). Thirty-seven patients (80.4%) suffered from class II dentoskeletal malformation and 8 had class III malformation (17%), while two patients (3%) had dentoskeletal class I with bimaxillary retrusion showed at the cephalometric analysis. The mean apnea-hypopnea index (AHI) of the group was 29.4 (SD: 12), and the mean body mass index (BMI) was 33.4 (SD: 5). All the patients were referred by neurology, otolaryngology, internal medicine, and pneumology specialists and were affected by OSAS with an underlying dentoskeletal malformation requiring surgical correction. All the patients underwent clinical assessment, teleradiography of cranium in two projections, CT scan of cranium without contrast, cephalometric analysis, and endoscopic assessment to properly study the air column of upper airways; both the clinical and instrumental assessments showed that orthognathic surgery (IIP procedure) was the best therapeutic option in all the cases.

All the patients were informed of the nature and indications of IP and IIP procedures as well as postoperative discomfort and possible complications. Postoperative discomforts presented as subdivided into two groups named “Group A” and “Group B”: the former encompassed all the postoperative discomforts resulting from both the IP and IIP procedures including pain, masticatory discomfort, need of analgesia, foreign body feeling, postoperative emesis, oronasal reflux, dysphagia, and edema of soft tissues. “Group B” discomforts were those related exclusively to IIP procedures and included possible temporomandibular dysfunction, aesthetic changes of the face, and possible damage of the third branch of the V cranial nerves.

We decided to compare some parameters of postoperative discomfort that were common to both the IP and IIP procedures, they included pain, painkiller need, admission days, foreign body sensation in the throat, and normal diet intake.

All parameters were recorded from the first postoperative day. None of the patients suffered from food intolerances or drug allergies. The postoperative prescriptions were the same for all the patients and included amoxicillin/clavulanic acid 2.2 gr i.v. every 12 hours and acetaminophen 1 gr i.v. if needed.

A Visual Analogic Scale (VAS) was used to objectivize the pain level, being 10 the value corresponding to maximum pain and 1 the condition of “no pain” [16, 17].

To evaluate the parameter “need of painkiller," the quantity of acetaminophen expressed in required doses was recorded and compared.

For the evaluation of the “foreign body sensation,” we also used a VAS assigning the value 10 to maximum foreign body feeling and 1 as normal feeling [16, 17]. The parameter “foreign body sensation” included the feeling of bulging in the pharynx as well as the oronasal reflux and postoperative dysphagia; it included also the postoperative emesis for patients who underwent IIP procedures.

The parameter “diet” entailed three degrees: normal diet, semisolid diet, and compulsory liquid diet.

To analyze the difference of postoperative discomfort between patients who underwent IP procedures and those who underwent IIP procedures, we divided the sample into two groups: on one side we pooled all the patients that accepted the surgical treatment either IP or IIP and defined that group as “surgery patients” (SP); the group “no surgery patients” (NSP) included all patients who refused surgery. The SP group was further subdivided into patients who accepted IIP procedures and those who agreed exclusively on IP procedures.

The SP group involved 28 patients; mean age was 39 years (SD: 11). Males were 17 (60.7% of the sample). Nine patients (32.1%) underwent I phase interventions. Among these 9 patients, 9 (100% of I phase group patients) underwent uvulopharyngopalatoplasty, 7 (77.8%) decongestion of turbinates and septoplasty, and 2 patients (22.2%) thyrohyoidpexy intervention. The remaining 19 subjects (67.9% of the whole sample) underwent II phase interventions (both Le Fort I osteotomy and bilateral sagittal osteotomy of the mandible); among those 19 patients, 5 (26.3% of the II phase group patients) had simultaneous genioplasty. Main demographic and clinical characteristics of the patients are summarized in Table 1.

To investigate the reason for refusing IIP procedures, we requested all participants belonging to NSP and IP procedures to indicate what was the group of postoperative discomfort that influenced their decision, choosing between “Group A” and “Group B.”

In the NSP group (18 patients, 39% of the sample), 5 were worried of postoperative discomforts belonging to “Group A” and 6 patients were discouraged by “Group B” discomforts, while 8 patients refused surgery for worries related to both “Group A” and “Group B” discomforts.

**Figure 1:**
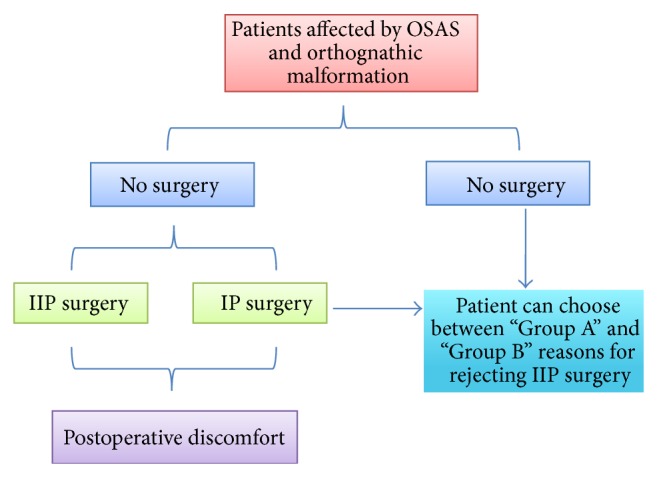
To investigate the motivations of refusing the IIP procedures, we requested all participants belonging to NSP and IP procedures to choose between “Group A” and “Group B” types of discomfort that influenced their choice.

Among the 9 patients who accepted only IP procedures, 6 patients refused IIP surgery because of “Group A” discomforts and 2 were concerned about “Group B” discomfort, while one patient indicated both “Group A” and “Group B” motivations (Figure 1).

Finally, to compare the real discomfort between IP and IIP procedures, the above cited parameters were analysed by Fisher's exact test and Student's *t*-test at days I, II, III, IV, V, VI, VII, XIV, XXI, and XXX; *P* < 0.005 was considered statistically significant.

## 3. Results

Statistically significant differences were observed between the study groups in terms of postoperative day at discharge (*P* < 0.001). I phase patients were discharged on average after 5.4 days, while II phase patients were discharged on average after 3.6 days (mean difference: 1.8). Differences in postoperative AHI were also statistically significant (crude *P* = 0.018; I phase mean postop AHI: 7.3, II phase mean postop AHI: 3.2; mean difference: 4.1), even after adjusting for preoperative AHI (adjusted *P* = 0.013).

**Figure 2:**
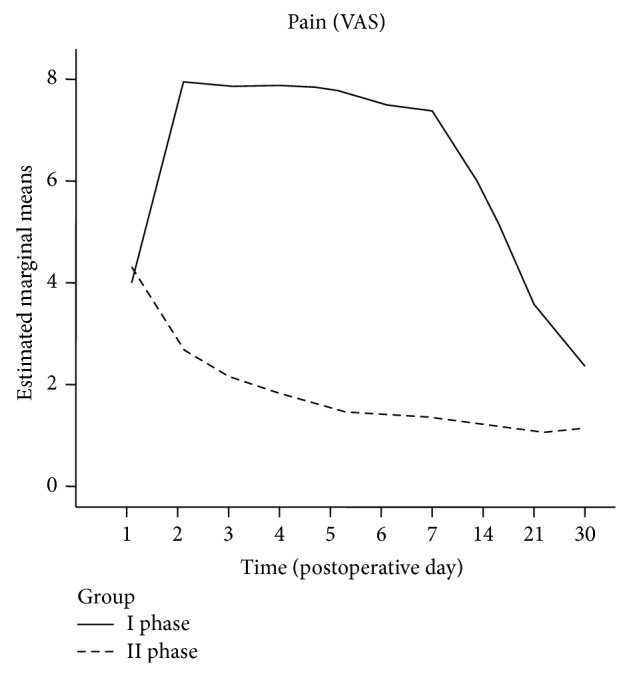
Postoperative pain. I phase versus II phase interventions.

Patients who underwent II phase interventions reported less pain on the Visual Analogic Scale and showed a better evolution of pain compared to those who underwent I phase interventions (see Figure 2).

**Figure 3:**
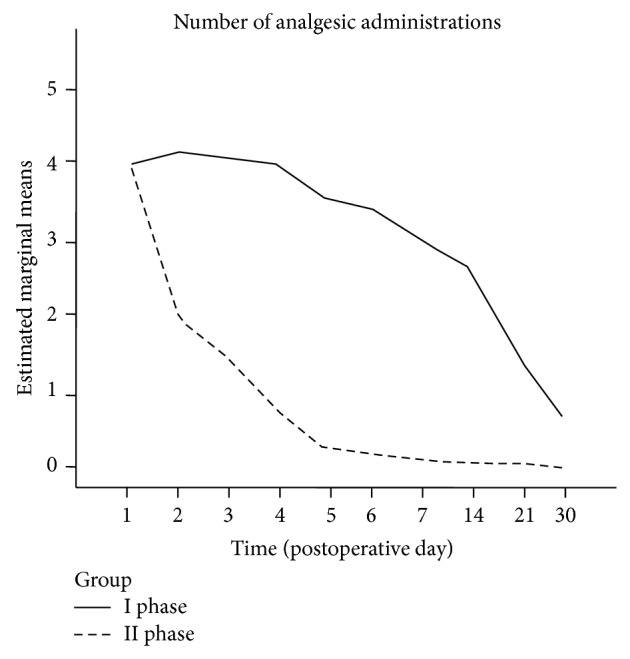
Number of postoperative analgesic administrations. I phase versus II phase interventions.

Between-groups effect of the type of intervention was statistically significant (adjusted *P* < 0.001) and also the interaction between time and intervention showed a between-groups significant effect (adjusted *P* = 0.001). Also the number of analgesic administrations was significantly lower in the II phase interventions group (between-groups effect of intervention, *P* < 0.001; between-groups effect of time-intervention interaction, *P* < 0.001; see Figure 3).

**Figure 4:**
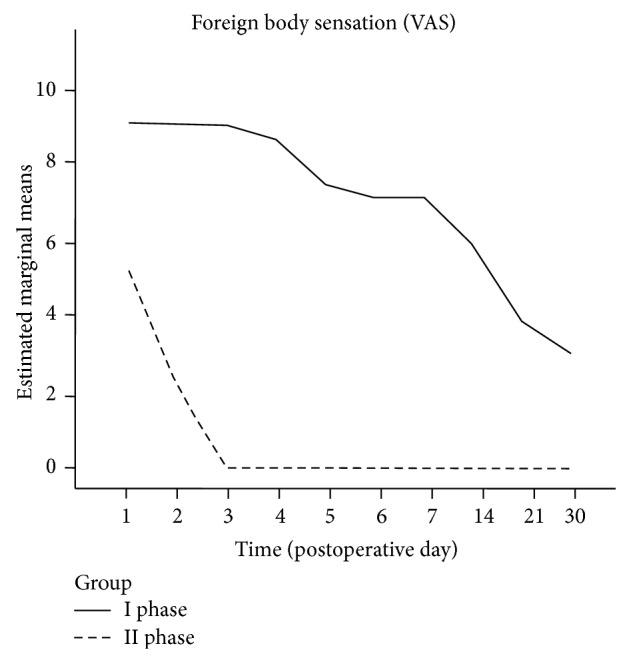
Postoperative foreign body sensation. I phase versus II phase interventions.

The foreign body sensation on the Visual Analogic Scale scored better among IIP patients, as both the between-groups effect of intervention (*P* < 0.001) and the between-groups effect of time-intervention interaction (*P* < 0.001) were statistically significant (see Figure 4).

Postoperative diet differed significantly between the groups during the whole analyzed postoperative period, as I phase patients could restart a normal diet before compared to II phase patients. Major findings are reported in Table 2.

## 4. Discussion

The relation between obesity and hypoventilation was first described in the late 1950's when obesity, hypopnea, and increased risks of heart diseases were positively correlated by several studies [18–23].

A careful depiction of the OSAS syndrome in the adult, in fact, can be actually found in Charles Dickens's novel* The Pickwick Paper*, afflicting the character Joe “the fat boy” [24].

The typical patients traditionally reported in literature were obese and in decayed physical conditions, and the elective treatment was a permanent tracheotomy; the outcomes were poor because of the high mortality and severe postoperative complications [25, 26].

The introduction of the continuous positive airway pressure (CPAP) as treatment for the OSAS in 1981 yielded positive outcomes for the first time [27]. The CPAP still remains the gold standard treatment for OSAS nowadays [11], although other therapeutic options have been proposed either as nonsurgical treatments by oral devices, neurostimulators, and drug-mediated therapies [28, 29] or by surgical interventions.

Besides the tracheotomy which, as mentioned, was the elective surgical procedure in the past [30], other surgical interventions to treat the OSAS were proposed starting from the early 1980's, mainly as result of the Stanford University group effort [31–33].

The surgical procedures proposed to treat OSAS were either directed exclusively to the soft tissues of the nose, rhino-, oro-, and hypopharynx (IP) or aimed at changing the position of the skeletal bases (IIP); usually IIP procedures were indicated after failure of IP interventions. Nowadays the most popular surgical treatment for OSAS is IP procedures, even though there is unanimous agreement that IIP surgery provides better and more reliable outcomes [7–15].

This study showed that the main reason discouraging the patients to accept IIP surgery was the concern of postoperative discomforts (Table 2).

Comparison of the parameter “postoperative pain” between patients who underwent IP procedures and those who had IIP surgery showed higher pain in the former group; patient treated with IP surgery reported severe pain for the first two postoperative weeks that was still present 30 days after surgery. The pain following IP surgery was typically sharp in nature, located at the throat and radiated to the ears; it is mainly due to the stimulation of free nociceptors (delta nervous fibers) on the raw surgical surfaces, the inflammatory status, and the muscular spasms [34]. The pain was described as continuous and exacerbated by deglutition (odynophagia); the odynophagia caused reduced oral intake and this condition promoted electrolytes imbalance and muscular spasms. The poor oral hygiene associated with the inflammation may favor the proliferation of the saprophyte oral bacteria inducing superinfection of the surgical wound and promoting exacerbation of both the inflammation and pain [35].

Second phase procedures (IIP), on the other hand, are performed through linear mucosal incision that are sutured and let to heal for first intention without exposure of terminal pain nerves; moreover, the subperiosteal exposure and the osteotomies contribute to temporary impairment of the function of the lower branch of the trigeminal nerve; as result, the postoperative pain following IIP surgery is nearly absent.

In our experience, patients who underwent IP procedures reported severe pain (VAS 7-8) during the first 14 postoperative days, with gradual decrease proportional to the healing of the surgical wounds; however, mild to moderate pain (VAS 2-3) was still recorded in this group at the 30th postoperative day. Patients who underwent IIP procedures, conversely, reported moderate pain (VAS 4) only on the 1st postoperative day and mild to no pain (VAS 0–2) starting from the 2nd postoperative day. None of the IIP patients complained of pain after the IV postoperative day. Our data are in accordance with what is reported in literature on postoperative pain rate following orthognathic surgery that is about 0.5% according to Politis et al. [36].

The need for painkiller is an indirect parameter of postoperative pain and our data are concordant with what is reported in the literature [35, 37]: IP patients required the maximum dosage of painkiller during the first 7 postoperative days; starting from the 14th postoperative day their need of painkiller was reduced to 2 doses a day. However, this group of patients required painkiller drugs until the 30th postoperative day, especially before sleeping.

The need for painkiller drugs in the IIP patient group reached its maximum at the 1st postoperative day to decrease steeply and come to a halt on the 3rd-4th postoperative day. The postoperative pain and need of painkiller influenced significantly the hospital admission time, which was longer (1.8 days more) in the IP group compared with the IIP group, negatively influencing the overall costs.

The parameter “foreign body sensation” showed different symptoms in IP and IIP procedures; generally IP procedures produce feeling of bulging palate combined with dryness of the pharynx because of the edema and reshaping of the soft palate and uvula, associated with the rearrangement of the nervous fibers and the excision of minor salivary glands. In our IP patients group, the foreign body sensation was severe in the first postoperative week (scoring 9 on VAS) and gradually decreased over the subsequent weeks to became mild (4 at VAS) on the 30th postoperative day. Our record is in accordance with what is reported in literature, where the feeling of foreign body following IP procedures is reported to gradually disappear in a timeframe of 6 to 12 months [1, 4, 16, 32, 38, 39].

Phase II procedure, namely, orthognathic surgery, was mainly burden by postoperative nausea and emesis as discomforts; Silva et al. [40] in 2006 outlined the positive correlation between pain and emesis and pointed out that maxillary surgery was strictly correlated with emesis.

Among the factors promoting nausea and emesis after orthognathic surgery we found the liquid diet, paresthesia/anesthesia of lips, orofacial edema, and blood swallowing during surgery. Combination of all those factors was associated with increased postoperative emesis following bimaxillary surgery [40, 41]. In our opinion, another factor implied in the postoperative emesis might be the changed relationship between the upper and lower dental arches; the new anatomical position of the jaws could be responsible for a foreign body feeling and promoting altered proprioceptive stimuli that will induce the emesis reflex.

In IIP patients, the VAS score for the emesis (indicated as foreign body sensation) was halved on the 2nd postoperative day and resolved (VAS = 0) starting from the 3rd postoperative day.

Regarding the “diet” parameter, we noticed different types of dysphagia in patients who underwent IP procedures with respect to those who underwent IIP surgery.

In IP procedures, the dysphagia was mainly due to the swallowing pain (odynophagia) of solid food; as already described, the inadequate oral intake determined a condition of undernutrition which triggered a vicious loop by inducing muscular spasms, which further exacerbated the pain and dysphagia. The symptoms usually improved with the healing of the surgical wounds, a process that takes several weeks during which there is a gradual return to a normal diet [35, 36].

Our IP patients had semiliquid diet for the first 14 days following surgery, avoiding spicy and acidic food; all the patients recovered a normal diet after 3 weeks postoperatively.

Patients who underwent IIP surgery assumed liquid diet for the first 30 days following surgery to avoid malunion or iatrogenic fractures of the osteotomized jaws; in the postoperative period, in fact, the altered muscular guide associated with a possible occlusal instability may predispose to iatrogenic fractures of the jaws if exposed to excessive masticatory burden. In our practice, we followed the international guidelines of intake and determined a condition of undernutrition which triggered a vicious loop by inducing muscular spasms, which further exacerbated the pain and dysphagia diet management after orthognathic surgery maintaining a liquid diet for 30 days, followed by further 30 days of blended diet before gradually reintroducing the normal diet [42].

## 5. Conclusions

The presented study showed that patients who underwent IP procedures suffered higher postoperative discomfort. On the light of this data, we believe that orthognathic surgery should not be a procedure to adopt in case of failure of the interventions of Phase I, but this should be the first choice, especially in cases with documented dental-skeletal malformations.

## Figures and Tables

**Table 1:** Main demographic and clinical characteristics of the patients enrolled in this study. I phase group versus II phase group.

	I phase (*N* = 9)	II phase (*N* = 19)	*P* value
Age, years [mean (SD)]	42 (12)	39 (11)	0.419^*^
Gender, males [*N* (%)]	7 (77.8)	10 (52.6)	0.249^**^
Dental class [*N* (%)]			
II	9 (100)	17 (89.5)	0.999^**^
III	0 (0)	2 (10.5)	
Preoperative AHI [mean (SD)]	30.6 (14.0)	28.4 (10.9)	0.691^*^
Postoperative AHI [mean (SD)]	7.3 (4.1)	3.2 (2.4)	0.018^*^
Preoperative BMI [mean (SD)]	32.3 (4.6)	31.8 (3.8)	0.767^*^
BMI at 6 months [mean (SD)]	29.7 (3.9)	29.2 (4.3)	0.796^*^
Postoperative day at discharge [mean (SD)]	5.4 (1.0)	3.6 (0.9)	<0.001^*^

^*^Student's *t*-test; ^**^Fisher's exact test.

**Table 2:** Postoperative diet at 7, 14, 21, and 30 days from the intervention. I phase group versus II phase group.

		I phase (*N* = 9)	II phase (*N* = 19)	*P* value
Day 7 [*N* (%)]	Normal	0 (0)	0 (0)	<0.001
	Soft food/semiliquid	8 (88.9)	0 (0)	
	Liquid diet	1 (11.1)	19 (100)	

Day 14 [*N* (%)]	Normal	2 (22.2)	0 (0)	<0.001
	Soft food/semiliquid	7 (77.8)	0 (0)	
	Liquid diet	0 (0)	19 (100)	

Day 21 [*N* (%)]	Normal	9 (100)	0 (0)	<0.001
	Soft food/semiliquid	0 (0)	0 (0)	
	Liquid diet	0 (0)	19 (100)	

Day 30 [*N* (%)]	Normal	9 (100)	0 (0)	<0.001
	Soft food/semiliquid	0 (0)	0 (0)	
	Liquid diet	0 (0)	19 (100)	
